# Exosomes in cancer metabolism and drug resistance: A review

**DOI:** 10.17305/bb.2025.13295

**Published:** 2025-11-05

**Authors:** Ousman Mohammed, Masresha Ahmed Assaye, Ermiyas Alemayehu, Abdisa Tufa, Solomon Genet

**Affiliations:** 1 Department of Medical Biochemistry, School of Medicine, College of Health Sciences, Addis Ababa University, Addis Ababa, Ethiopia; 2 Department of Medical Laboratory Sciences, College of Medicine and Health Sciences, Wollo University, Dessie, Ethiopia; 3 Department of Internal Medicine, School of Medicine, College of Health Sciences, Addis Ababa University, Addis Ababa, Ethiopia

**Keywords:** Exosomes, tumor microenvironment, metabolic reprogramming, drug resistance, immune evasion, intercellular communication, metastasis

## Abstract

The transfer of molecular cargo in exosomes plays a crucial role in cancer progression, influencing metabolic processes, angiogenesis, immune interactions, and invasive capabilities. This review synthesizes current evidence on how exosomes modulate tumor metabolism and drive drug resistance, and outlines therapeutic opportunities. We searched PubMed, Scopus, Web of Science, and Google Scholar for English-language studies using terms related to exosomes/extracellular vesicles, glycolysis, oxidative phosphorylation (OXPHOS), lipid metabolism, and drug resistance/chemoresistance, and integrated the literature qualitatively. Evidence indicates that exosomes reprogram tumor and stromal metabolism by delivering enzymes and non-coding RNAs that boost glycolysis and dampen OXPHOS, activate cancer-associated fibroblasts and extracellular matrix (ECM) remodeling, and modulate ferroptosis. They stimulate angiogenesis (e.g., via vascular endothelial growth factor (VEGF)/Wnt pathways) and promote immune escape through programmed death-ligand 1 (PD-L1), transforming growth factor beta (TGF-β), and macrophage reprogramming. Exosomal integrins and proteases contribute to epithelial–mesenchymal transition (EMT), organotropism, and pre-metastatic niche formation. Critically, exosomes propagate chemoresistance by exporting drugs and spreading determinants—including P-gp/BCRP/MRP-1, anti-apoptotic proteins, and regulatory RNAs—to previously sensitive cells; adipose-derived vesicles and lipid cargos further reinforce metabolic plasticity and therapy resistance. Given their stability, nanoscale dimensions, and ability to cross the blood–brain barrier, exosomes are promising vectors for targeted delivery; engineered vesicles can enhance chemotherapy responsiveness and counteract resistance, particularly alongside immunotherapy. In summary, interventions that disrupt exosome biogenesis, cargo loading, or uptake—paired with engineered exosomes for precision delivery—could mitigate drug resistance, metastasis, and immune evasion and advance more effective cancer treatment.

## Introduction

Cancer development is influenced by various factors, including genetic mutations and the pivotal role of the tumor microenvironment (TME) [1]. Interactions within the TME significantly contribute to cancer-associated metabolic reprogramming, emphasizing the importance of intercellular communication in supporting tumor growth [2, 3]. To survive in nutrient-deprived conditions, cancer cells adapt their metabolism to extract essential nutrients, thereby promoting cell viability and proliferation [4]. As first observed by Otto Warburg in the 1920s, cancer cells preferentially utilize glycolysis over oxidative phosphorylation (OXPHOS), resulting in increased glucose uptake and rapid, albeit less efficient, energy production [5]. This metabolic shift also provides precursors necessary for the synthesis of lipids, amino acids, and nucleotides, all of which are vital for rapid tumor growth [6, 7].

**Figure 1.**
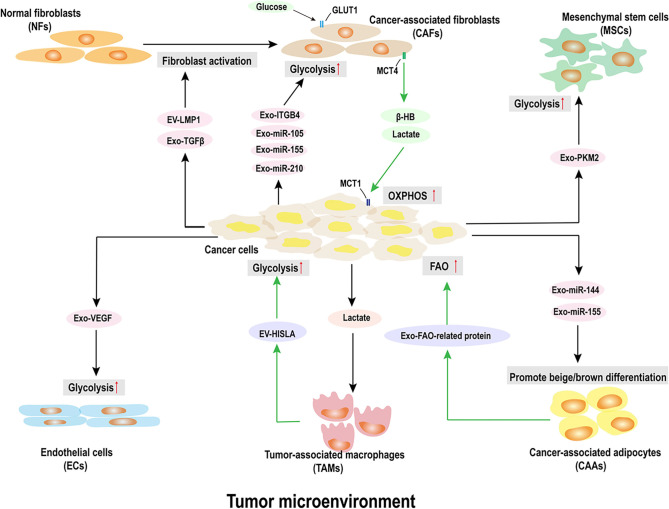
**Exosomal cargo in cancer progression.** Adapted from Yang et al. (2025) [3]. Exosomes play a crucial role in tumor growth, angiogenesis, metastasis, treatment resistance, and immunosuppression. They enhance glycolysis in cancer cells, weaken the endothelial cell barrier, and modify metabolic processes, thereby facilitating tumor dissemination. Drug-resistant exosomes can be incorporated by drug-sensitive cells, resulting in a phenotypic transformation of the cancer cells.

Previously regarded as mere cellular waste, exosomes are now recognized as critical mediators in cancer progression. These small extracellular vesicles (EVs) carry a range of bioactive molecules—including proteins, lipids, non-coding RNAs (ncRNAs), and metabolites—originating from both nuclear and mitochondrial sources [8–11]. Tumor-derived exosomes are often enriched with oncogenic components that promote tumor growth, invasion, and immune evasion. For instance, they can convert fibroblasts into cancer-associated fibroblasts (CAFs) through glycolytic signaling. These CAFs subsequently release metabolites such as lactate and exosomes containing nutrients that further support cancer cell metabolism. Similarly, immune cell-derived EVs can deliver RNAs that enhance glycolysis in tumor cells, contributing to immune escape (Figure 1) [12, 13].

Exosomes also play a significant role in chemoresistance. They can transfer anti-apoptotic proteins and miRNAs to neighboring cancer cells, altering their phenotype and enhancing drug resistance. Moreover, exosomes facilitate the direct removal of chemotherapeutic agents such as doxorubicin, cisplatin, and paclitaxel through vesicle shedding [3, 14–17]. Additionally, they disseminate functional drug efflux transporters, including P-glycoprotein (P-gp), BCRP, and MRP-1, to drug-sensitive cells, thereby propagating resistance traits throughout the tumor population [3, 18, 19]. This dual mechanism not only enhances drug clearance but also enables the horizontal transmission of resistance features.

Beyond chemoresistance, exosomes contribute to metastasis by transporting factors that promote metastasis, reprogramming stromal cells, and modulating immune responses to create pre-metastatic niches [9]. They reshape the extracellular matrix (ECM), establishing a supportive microenvironment that fosters tumor survival and expansion under various stress conditions. Given their distinct molecular cargo across different tumor types, exosomes present promising clinical potential as diagnostic biomarkers and therapeutic targets [15, 16].

Understanding the multifaceted roles of exosomes in the TME—including their influence on metabolism, metastasis, and drug resistance—could pave the way for innovative cancer therapies. Our search methodology includes databases (Google Scholar, PubMed, Scopus, and Web of Science) utilizing search criteria (exosomes/EVs, glycolysis/OXPHOS/lipid metabolism, and drug resistance/chemoresistance), along with a restriction to English-language studies. This review discusses the central roles of exosomes in tumor progression and therapy resistance, highlighting their value in the development of next-generation cancer treatment strategies.

## Unveiling exosomes: Architecture, molecular makeup, and biogenesis

The term “EVs” encompasses all membrane-bound vesicles released by cells, including exosomes and microvesicles. EVs are typically classified based on their physical and biochemical characteristics, as well as their size, with small EVs being less than 200 nm and medium/large EVs exceeding 200 nm [16]. Despite advancements in exosome isolation technologies, including ultracentrifugation, size-exclusion chromatography, and precipitation, challenges persist due to contamination from protein aggregates, lipoproteins, and cell debris. Furthermore, no single molecular marker uniquely identifies exosomes; instead, a combination of markers (e.g., CD9, CD63, CD81, TSG101, ALIX) is recommended to confirm EV enrichment [16, 20, 21]. Due to these methodological constraints, caution is warranted when interpreting EV studies, underscoring the need for consistent experimental and reporting standards.

Exosomes are lipid bilayer-enclosed vesicles (30–150 nm) released into the extracellular fluid [16, 20]. They carry diverse biomolecules essential for cellular function, playing a critical role in biogenesis. Exosomes also contain heat shock proteins for protein stability, transport proteins for vesicle trafficking, and cytoskeletal elements. Additionally, the presence of metabolic enzymes underscores their functional versatility [21–23]. The process of exosome development involves initiation, secretion, formation of multivesicular bodies (MVBs), and endocytosis, with early endosomes evolving into MVBs containing intraluminal vesicles destined for degradation or release [24, 25]. Some exosomes, characterized by lysosomal-associated membrane proteins 1 and CD63 markers, are released into the extracellular space, while others are directed towards lysosomal degradation [26, 27]. In tumors, exosome release involves MVB trafficking, docking, and fusion, driven by cytoskeletal elements, motor proteins, and the SNARE complex. Small GTPases regulate vesicle dynamics [28, 29]. Notably, GTPases such as Rab5, Rab27, Rab35, RalA, and RalB play essential roles in regulating vesicle dynamics; particularly, Rab27a and Rab27b are crucial for exosome secretion, with Rab27 inhibition demonstrating potential to reduce exosome release and impede tumor progression [29]. Exosomes impact target cells through three primary mechanisms: direct receptor interaction, membrane fusion releasing cargo into the cytoplasm, and internalization via phagocytosis or endocytosis [30, 31].

## Role of exosomes in TME and metabolic reprogramming

### Cancer-derived exosomes and CAFs

Exosome production is significantly elevated in cancer, with heightened levels detected in the body fluids of patients. This increase in secretion is driven by factors such as hypoxia, p53 mutations, and Rab GTPases [32]. Hypoxia is frequently present in TMEs, contributing to hypoxia-driven cancer growth. Exosomes derived from hypoxic tumor cells are enriched with matrix metalloproteinase-13 and microRNA-21, which promote metastasis by inducing epithelial–mesenchymal transition (EMT)—increasing vimentin expression while downregulating E-cadherin in normoxic cells. The hypoxia-inducible factor 1-alpha (HIF-1α) activates the rapid accumulation of these genes [32, 33]. As tumors progress, fibroblasts undergo substantial changes, acquiring activated characteristics and enhancing the production of collagen and hyaluronic acid. This activation is often mediated by cancer-derived exosomes via the transforming growth factor beta (TGF-β)/Smad signaling pathway [34, 35]. Exposure to tumor-derived EVs (TDEVs) in CAFs induces premalignant characteristics in normal epithelial cells, promoting their proliferation and oncogenic factor expression, and transforming fibroblasts into CAFs [36, 37].

Reprogrammed CAFs release exosomes that further influence cancer cells by enhancing metabolic shifts and remodeling the ECM, thereby facilitating tumor development [38]. These exosomes contain factors such as stromal cell-derived factor 1 (SDF-1), matrix metalloproteinase-2 (MMP-2), and transforming growth factor beta, which collectively drive tumor initiation and progression through diverse interactions within the TME [38, 39]. Additionally, exosomal miRNAs encourage the transformation of healthy fibroblasts into CAFs while aiding immune evasion, underscoring their pivotal role in cancer progression [17, 38]. Specific miRNAs, such as miR-630 and miR-210, identified in cancer-derived exosomes from ovarian, liver and lung cancers, drive the conversion of fibroblasts into CAFs via the NF-κB and Janus kinase 2 pathways [40--43]. Moreover, exosomal regulation of miRNAs, including miR-31, miR-105, miR-214, and miR-155, facilitates the transformation of fibroblasts into CAFs and enhances immune evasion [41–43].

**Figure 2.**
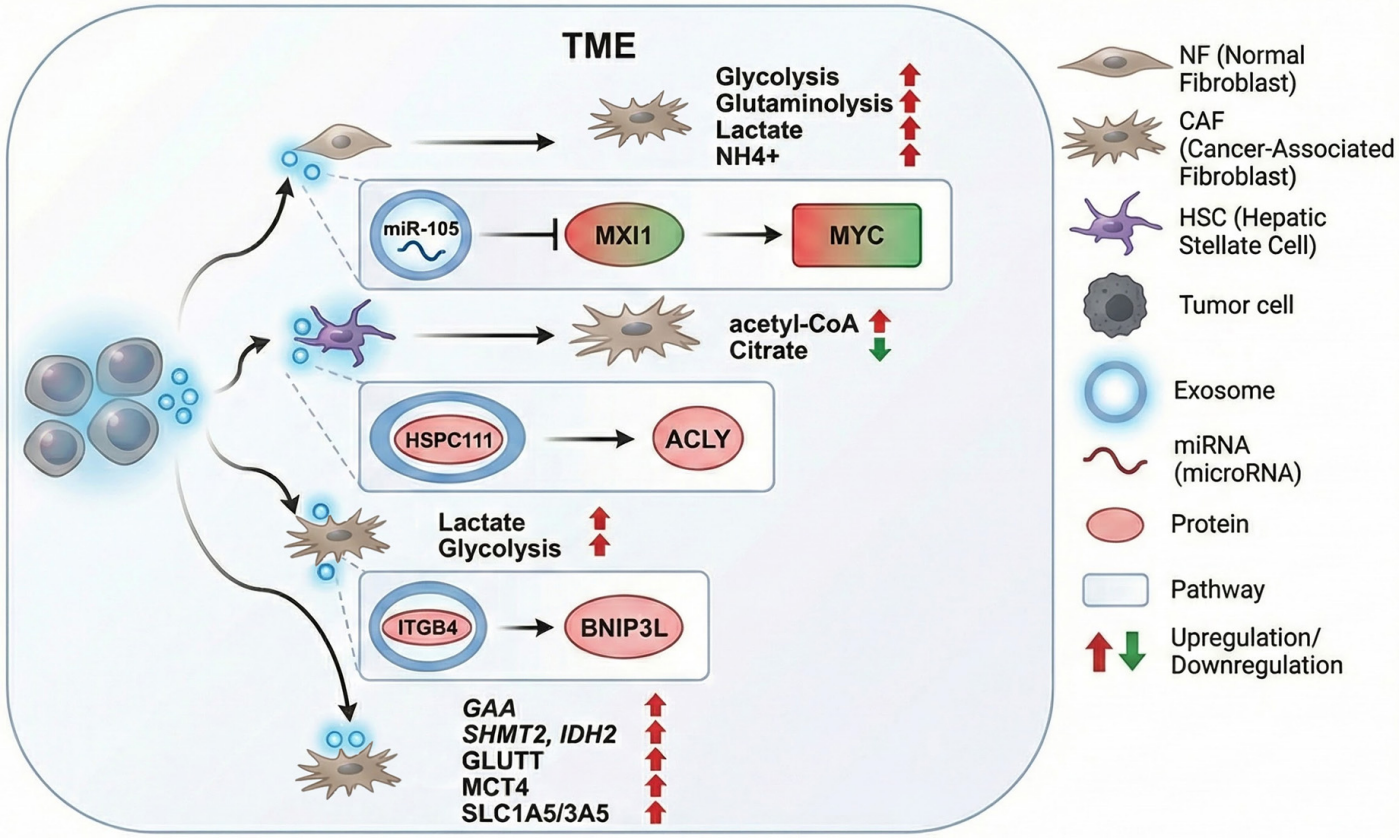
**Mechanisms of metabolic reprogramming in the Tumor Microenvironment (TME) mediated by cancer-derived exosomes.** Tumor cells secrete exosomes that facilitate intercellular communication, reprogramming stromal components---specifically Normal Fibroblasts (NFs) and Hepatic Stellate Cells (HSCs)---into a pro-tumorigenic Cancer-Associated Fibroblast (CAF) phenotype. This transformation is driven by three primary exosomal signaling axes: (A) Exosomal miR-105 downregulates MAX interactor 1 (MXI1), releasing the suppression of MYC. MYC activation subsequently upregulates glycolysis, glutaminolysis, and lactate production while promoting ammonia (NH 4 + detoxification. (B) Exosomal HSPC111 (NOP16) activates ATP-citrate lyase (ACLY) in HSCs, leading to increased Acetyl-CoA production and facilitating lipid metabolic rewiring. (C) Exosomal Integrin β4 (ITGB4) induces BNIP3L expression, promoting mitophagy and glycolysis (Warburg effect). The convergence of these pathways results in the upregulation of key metabolic enzymes and transporters (including GLUT1, MCT4, and IDH2), creating a nutrient-rich niche that supports tumor proliferation. Adapted from Li et al., 2020 [52].

CAF-derived exosomes (CDEs) modulate fatty acid (FA) metabolism to support lipid synthesis, which is crucial for cell membrane formation and energy storage [33, 44–46]. CDEs enhance FA metabolism by increasing acetate incorporation, promoting glutamine-driven synthesis, and repressing glucose oxidation for lipid generation [47]. They transport key metabolites, such as acetyl CoA and essential enzymes involved in lipid synthesis and the tricarboxylic acid (TCA) cycle, thus influencing the metabolism of recipient cells [48, 49]. CDEs serve as vital sources of metabolites that fuel the TCA cycle and sustain cancer cell proliferation under nutrient-deprived conditions (Table 1) [33]. In pancreatic cancer, CDEs rich in amino acids and TCA intermediates sustain cellular proliferation under nutrient stress. Elevated levels of citrate and pyruvate in exosomes further highlight their role in enhancing metabolic activity [11, 33, 47]. CAFs contribute to tumor progression through the reverse Warburg effect, leading to glucose breakdown and lactate export, a process amplified by the upregulation of key enzymes and transporters (Figure 2) [50–52].

**Table 1:** Integrated framework linking extracellular vesicle cargo, metabolic reprogramming, resistance phenotypes, and tumor context

**Exosomal cargo class**	**Primary metabolic reprogramming**	**Resistance/Phenotype**	**Exemplar tumor type**	**Ref.**
Glycolytic enzymes and ncRNAs (HK2, PKM2, LDHA; circ-RNF121, MALAT1, TUG1)	Glycolysis upregulation	Anti-apoptosis, EMT, proliferation	Colorectal, NSCLC, HCC	[33, 41, 60–63]
Glucose transporters (GLUT1/GLUT3)	Glucose uptake	Chemoresistance, lactate production	Lung adenocarcinoma, colorectal	[54, 55]
Mitochondrial regulators (PGC-1α, CYTB, COXI; miR-22, SNHG3)	OXPHOS suppression	Metabolic rewiring, drug resistance	Breast, colorectal	[33, 53]
Lipid metabolism enzymes and ncRNAs (ACLY, FASN, miR-122, NOP16/HSPC111)	Fatty acid synthesis/storage	Energy adaptation under stress	Breast, colorectal	[44–46, 52]
Ferroptosis modulators (ALOX15, miR-522, miR-1246)	Lipid peroxidation/iron homeostasis	Ferroptosis resistance, immune evasion	Gastric, HCC	[36, 57, 58]
Immune-modulatory ncRNAs/proteins (miR-21, miR-1246, PD-L1, FasL, TGF-β)	Immune checkpoint/signaling	Immune evasion, M2 macrophage polarization	NSCLC, ovarian, pancreatic	[88–93]
Exosomal integrins and adhesion proteins (ITGB4, LMP1)	ECM and signaling	Anti-apoptosis, metastatic niche formation	Breast, nasopharyngeal carcinoma	[52, 65]

Abbreviations: ncRNAs: Non-coding RNAs; EMT: Epithelial–mesenchymal transition; OXPHOS: Oxidative phosphorylation; ECM: Extracellular matrix; HCC: Hepatocellular carcinoma; NSCLC: Non-small cell lung cancer.

CDEs inhibit OXPHOS in tumor cells by downregulating mitochondrial genes such as CYTB and COXI, a process mediated by specific miRNAs, including miR-22, let-7a, and miR-125b [33]. Tumor-derived exosomes activate HIF-1α, which increases glycolytic enzymes and proteins such as HK2 and GLUT1. Furthermore, long ncRNAs (lncRNAs) like SNHG3 sequester miR-330-5p, promoting PKM expression to inhibit OXPHOS [53]. In colorectal cancer, CDEs upregulate GLUT1 via caveolin-1, enhancing glucose uptake [54]. In lung adenocarcinoma, exosomal LINC01614 stimulates glutamine addiction and glycolysis through the NF-κB pathway [55]. Through the miR-524-5p/SIX1 axis, lncRNA TUG1 promotes glucose metabolism in hepatocellular carcinoma (HCC) [56]. Additionally, CDEs foster an inflammatory TME, as observed in HCC, where exosomal miR-1247-3p activates interleukin release via β1-integrin and NF-κB activation [57]. Gastric tumor cells secreting exosomal miR-522 prevent ferroptosis by targeting ALOX15 [58].

Exosome-mediated communication within the TME influences ferroptosis, a metabolism-regulated form of cell death characterized by an imbalance between oxidative and antioxidant systems. Exosomes can either promote or inhibit ferroptosis, thus affecting tumor reprogramming, the immunosuppressive microenvironment, and pre-metastatic niche development. This regulatory capacity impacts cancer cell behavior and drug sensitivity. Exosomes from cancer cells can protect stromal cells from ferroptosis, while exosomes from stromal cells can induce ferroptosis resistance, thereby promoting cancer growth. Understanding these mechanisms will enhance the development of targeted therapies for malignant tumors [36].

### Exosomal ncRNAs in cancer metabolic reprogramming

The mechanisms underlying the encapsulation of lncRNAs in exosomes remain poorly understood; however, studies suggest that proteins such as HuR and hnRNPA2B1 facilitate the sorting of lncRNAs into exosomes. RNAs exist within cells or exosomes as ribonucleoprotein complexes, necessitating the involvement of proteins that form complexes with RNAs for encapsulation [40]. Exosomal ncRNAs, characterized by low rRNA content, regulate malignant cell properties, impacting immune evasion and cellular proliferation. These RNAs are shielded from degradation by the exosomal membrane, allowing them to influence cellular growth, tissue remodeling, and immune system evasion [59]. Exosomal circ-RNF121 promotes glycolysis and tumor size increase in colorectal cancer [60]. In certain lung cancers, exosomal circ-0008928 drives cisplatin resistance by sequestering miR-488, thereby enhancing hexokinase 2 (HK2) activity [61]. Exosomal lncRNAs also facilitate glycolytic processes; for instance, lncRNA MALAT1 in non-small cell lung cancer (NSCLC) upregulates lactate dehydrogenase A (LDHA) [61], while circ-0005963 enhances pyruvate kinase (PKM2) activity by sequestering miR-122 in colorectal cancer [62]. In HCC, lncRNA TUG1 secreted by altered fibroblast cells increases LDH activity and lactate levels, thus promoting tumor progression [41].

In glioma cells resistant to temozolomide (TMZ), circ-0072083 is secreted, elevating glucose transporter and glycolytic enzyme levels, thereby reinforcing both glycolysis and treatment resistance [63]. In melanoma, exosomes enriched with miR-155 and miR-210 amplify glycolysis under normoxic conditions while reducing OXPHOS in fibroblasts, contributing to extracellular acidification [64]. Exosomes from malignant cells containing miR-105 enhance glucose and glutamine metabolism in fibroblasts [65]. Exosomal miRNAs from nasopharyngeal cancer activate aerobic glycolysis and autophagy in tissue cells through the LMP1-NF-κB/p65 signaling pathway, thereby exacerbating hypoxia within the TME [50].

Under hypoxic conditions, tumor-derived exosomes transfer specific microRNAs, such as let-7a and miR-21-3p, to macrophages [66–68]. Hypoxia also stimulates the release of exosomes regulated by HIF-1α. Exosomal microRNA-310a-5p inhibits proline hydroxylase, preventing HIF-1α degradation and promoting malignant cell growth, dissemination, and EMT, all of which are key drivers of cancer progression. Furthermore, lung cancer-derived exosomes exhibit elevated levels of miR-23a under hypoxic conditions, which inhibits proline hydroxylase enzymes in endothelial cells (ECs) [69]. Similarly, microRNA-142-5p is transported by exosomes to ECs, resulting in the expression of indoleamine 2,3-dioxygenase (IDO) protein, leading to CD8+ T cell suppression and exhaustion [70].

### Exosomal proteins in cancer progressions

Exosomal proteins play a crucial role in modifying glucose metabolism in cancer cells, facilitating rapid growth and survival under adverse conditions. Exosomes derived from stromal cells transport glucose transporters and PKM2, thereby enhancing glycolytic activity in dormant cells. In contrast, exosomes from tumor cells carry metabolic enzymes involved in the glycolysis pathway, promoting glycolysis, tumor dissemination, and resistance to treatment in adjacent cells [17, 71, 72]. Additionally, exosomes harbor angiogenic and metabolic factors like VEGF and integrin beta 4 (ITGB4), which contribute to the reprogramming of stromal cells. Different exosomes secreted by cancer cells are rich in hexokinase and PKM2, accelerating glycolysis in target cells [73].

Exosomes containing latent membrane protein 1 (LMP1) can convert fibroblasts into tumor-supportive phenotypes by upregulating glycolysis and suppressing mitochondrial functions via the NF-κB pathway, thereby reshaping the TME to promote growth and immune evasion [50]. Similarly, exosomes from triple-negative breast cancer (TNBC) contain ITGB4, which induces mitophagy in fibroblasts, further promoting glycolysis and tumor growth [74]. Exosomes derived from lung adenocarcinoma that contain high mobility group box 1 enhance glucose metabolism in tumor-associated macrophages (TAMs) through programmed death-ligand 1 (PD-L1) upregulation, thereby fostering an immunosuppressive environment [75]. Furthermore, exosomes from cancer cells transfer zinc transporter protein 4 (ZIP4), enhancing the malignancy of less aggressive cancer cells by reprogramming metabolic and proliferative pathways [65]. Thus, exosomal proteins function as versatile mediators of cancer progression, central to altering metabolism in malignant cells [23].

### Exosomal cargos’ function in inducing angiogenesis

Angiogenesis is a significant driver of tumor growth, and tumor-derived exosomes are essential for the formation of premetastatic niches. Exosomes from metastatic tumors stimulate angiogenesis by inducing Ephrin-B reverse signaling, while exosomes loaded with miR-210 and annexin II (ANX II) also promote angiogenesis [76]. Exosomes facilitate the transfer of information between endothelial and cancer cells, thereby inducing angiogenesis and enhancing vascular permeability, which ultimately contributes to metastasis. ECs, as primary mediators of angiogenesis, migrate and proliferate to form new blood vessels, creating pathways that facilitate premetastatic niche formation and accelerate cancer cell dissemination [77]. Cancer-derived exosomes enhance VEGF secretion from human umbilical vein ECs through mechanisms involving exocirc-0044366 (circ29), which acts as a miR-29a sponge, inhibiting its binding to VEGF mRNA, and circSHKBP1, which stabilizes VEGF mRNA by promoting the expression of human antigen R (HuR) [78]. Exosomes from gastric and hypoxic cancer cells contain Y-box binding protein-1, which promotes angiogenic factors such as IL-8 and MMP-9 and transmits Wnt4, facilitating β-catenin nuclear translocation [79]. Moreover, exosomal miR-21-5p enhances this angiogenic process by promoting β-catenin translocation, thereby amplifying the effects of the pathway [80].

Exosomes derived from endothelial progenitor cells (EPCs) deliver miR-210, which reduces reactive oxygen species (ROS), enhances ATP production, and stabilizes ECs [52]. In acute myeloid leukemia (AML), exosomes carrying VEGFR mRNA increase VEGF receptor expression in ECs, promoting glycolysis, vascular remodeling, and chemoresistance [81]. Tumor-derived exosomes transport enzymes such as CD39 and CD73, which hydrolyze extracellular ATP, generating adenosine that stimulates EC growth, angiogenesis, and tumor progression [52]. These exosomes also induce macrophages to adopt an angiogenic phenotype (M2 polarization), which leads to angiogenesis through the production of angiopoietin-1 and endothelin-1 [18, 82]. Cancer-derived exosomes encapsulate IL-6, IL-8, and microRNAs such as miR-205, which activate the AKT pathway by inhibiting PTEN, thereby supporting angiogenesis in chemoresistant cancers [83]. Transcription factors, such as ATF2 and MTA1, present in cancer-derived exosomes, drive both angiogenesis and metastasis [63]. Exosomes from pancreatic cancer cells, carrying miR-1290 and Lin28B, activate pancreatic stellate cells (PSCs), stimulating the production of VEGF, fibroblast growth factor (FGF), and MMPs, thereby promoting vascular development and tumor growth [84, 85].

### Exosomes regulate immune cell function

Exosomes released by immune cells, mesenchymal stem cells, and platelets play a critical role in regulating immune cell metabolism in malignancy, influencing immune responses and tumor progression [46, 86, 87]. In the early stages of tumor growth, activated immune cells release exosomes that enhance antitumor responses; however, as tumors progress, they produce exosomes with immunosuppressive properties that inhibit antitumor immune cell activity and promote tolerance through myeloid-derived suppressor cells (MDSCs). These cancer-generated exosomes significantly shape the immune landscape, facilitating immune evasion [88–90].

Tumor-derived exosomes carry immunosuppressive factors such as Fas ligand (FasL), PD-L1, and TGF-β1. Cancer-secreted exosomes enriched with microRNA-21 shift monocytes toward an immunosuppressive M2 phenotype [23, 48]. In cancer, p53-mutant cell-derived exosomes release miR-1246, which reshapes TAMs to enhance TGF-β secretion and promote tumor growth [91–94]. TAM-derived exosomes contain long non-coding RNA (HISLA), which stabilizes HIF-1α and promotes oxyglycolysis in cancer cells [95]. In pancreatic cancer, exosomes from SMAD4-deficient cells shift immune cells toward glycolysis and increased calcium signaling, further promoting an immunosuppressive state [96]. A study indicates that TAMs promote aerobic glycolysis and resistance to apoptosis in malignant cells by transferring the lncRNA HISLA through exosomes [97]. In gliomas, TAMs secrete IL-6, promoting PDPK1-mediated phosphorylation of PGK1 at threonine 243, which enhances aerobic glycolysis and tumor growth [98].

TAM-derived exosomal DOCK7 reduces intracellular cholesterol by activating RAC1 and ABCA1 through the AKT/FOXO1 pathway, thereby increasing membrane fluidity, cell motility, and invasiveness [99]. In HCC, TAM-derived exosomes deliver lncRNA RP-11-1100L38 (lncMMPA), downregulating aldehyde dehydrogenase 1 family member A3, increasing lactate production, and promoting tumor cell proliferation [100]. Similarly, TAM exosomes are critical to cancer aggressiveness by modifying gene expression: miR-95 targets JunB in prostate cancer, driving proliferation and EMT; miR-221-3p inhibits CDKN1B in epithelial ovarian cancer, fostering cell cycle progression, and tumor growth [101]. In gastric cancer, TAM-derived miR-21 targets PDCD4 to enhance proliferation and inhibit apoptosis [102]. Conversely, liposarcoma exosomes containing miR-25-3p and miR-92 activate TLR7/8 in TAMs, stimulating IL-6 production and a pro-inflammatory response [103]. Exosomes not only promote tumor progression but also hold potential as anticancer vaccines. They deliver tumor-associated antigens (TAAs) and MHC molecules, enhancing CD8+ T cell responses and stimulating antitumor immunity [104].

Cancer-derived exosomes mediate immune suppression by transporting ligands, proteins, and miRNAs that inhibit T cells [32]. For instance, exosomal PD-L1 from melanoma reduces CD8+ T cell activity [105, 106], while exosomes polarize macrophages toward an M2 phenotype via pathways such as PI3K/AKT and STAT3, promoting the release of immunosuppressive molecules [107]. Another mechanism of immune inhibition involves ATP hydrolysis by the nucleotidases CD39 and CD73, leading to increased cytosolic cAMP levels and suppression of immune functions [108]. Mammary carcinoma-derived exosomes rich in prostaglandin E2 and TGF-β heighten MDSC expansion and VEGF production via the MyD88 signaling pathway [109]. Exosomes also suppress dendritic cell (DC) growth and T cell responses, facilitating their evasion. Pancreatic cancer-derived exosomes upregulate PD-L1 in macrophages, inhibiting TLR4 expression in DCs [110]. Furthermore, TAM-derived exosomes carrying miR-29a-3p and miR-21-5p disrupt the Treg/Th17 balance, further facilitating tumor immune escape and metastasis [111].

### Adipose tissue-derived exosomes in cancer

Cancer cells and adjacent adipocytes engage in bidirectional lipid exchange, which accelerates tumor growth. Adipocyte–adipocyte interactions facilitate FA transfer and lipolysis, leading to β-oxidation in cancer cells [76]. Exosomes derived from adipose tissue play a critical role in supporting cellular functions, thereby contributing to cancer spread and metastasis. Adipocytes within the TME significantly influence tumor growth through metabolic reprogramming and secretory mechanisms, often modulated by exosomal cargo derived from cancer cells [112]. The lipid composition of exosomes differs from that of parental adipocytes, featuring membranes enriched with SM, gangliosides, and saturated FAs. These exosomes exhibit a more PS-rich outer leaflet, with phosphorylated phosphatidylethanolamine (PE) distributed between the two membrane layers. Additionally, exosomes contain lipid metabolism enzymes, including phospholipase A2 (PLA2) classes, which efficiently transport bioactive lipids, potentially enhancing tumor development, metastasis, and immune suppression [76, 113].

Adipose tissue-derived exosomal miR-155 promotes the differentiation of beige and brown adipocytes by targeting PPARγ, while miR-126 influences lipid accumulation and glucose transport via the insulin receptor substrate/GLUT-4 signaling pathway. Exosomes produced by adipocytes primarily enhance the transfer of FAs rather than enzymes related to FA oxidation. In HCC characterized by high body fat percentages, elevated levels of miR-23a and miR-23b in blood exosomes promote tumor growth and chemoresistance by modulating the hypoxia-inducible factor-1 (HIF-1) signaling pathway [113]. In lung cancer, hypoxic adipocyte-derived exosomes reduce miR-433-3p levels, resulting in increased expression of stearoyl-CoA desaturase 1, which supports tumor cell proliferation and lipid accumulation [114].

Exosomes from obese adipose tissue enhance mitochondrial density, oxygen consumption rates, and ATP production in MCF-7 cells, promoting OXPHOS-dependent proliferation. These exosomes can influence tumor metabolism by enhancing OXPHOS, activating the AKT/mTOR/P70S6K signaling pathway, inhibiting ferroptosis, increasing FA oxidation, and modulating mitochondrial dynamics. They induce a metabolic shift toward glycolysis, thereby promoting tumor aggressiveness and resistance to docetaxel [3, 59]. Furthermore, adipocyte-derived exosomes enhance chemoresistance and tumor migration by transferring microsomal triglyceride transfer protein (MTTP), which forms a complex with proline-rich acidic protein 1 (PRAP1). This interaction inhibits the ZEB1 transcription factor and stimulates glutathione peroxidase 4 and the cystine/glutamate antiporter, thereby reducing lipid peroxidation and susceptibility to ferroptosis. Knockdown of MTTP in obese mice increases sensitivity to oxaliplatin, highlighting its role in chemoresistance (Table 2) [115].

**Table 2:** The role of exosomal cargo in cancer progression and tumor microenvironment modulation

**Theme**	**Mechanism**	**Exosomal cargo**	**Effects on TME or cancer cells**	**Ref.**
CAF activation and metabolic reprogramming	Induction of CAF phenotype in fibroblasts	**Proteins:** TGF-β RNAs: miR-630, miR-210, miR-155, miR-214, miR-105	Activates TGF-β/Smad, NF-κB, and JAK2 signaling; promotes ECM remodeling and immune evasion	[32–43]
	CAF-derived exosomes support cancer metabolism	**Metabolites:** acetyl-CoA, citrate, pyruvate, TCA cycle intermediates	Enhances lipid biosynthesis and metabolic adaptation under nutrient stress (reverse Warburg effect)	[33, 44–52]
	Suppression of mitochondrial activity in cancer cells	**RNAs:** miR-22, let-7a, SNHG3, TUG1	Downregulates CYTB and COXI; upregulates glycolytic proteins (HK2, GLUT1, PKM)	[33, 53–56]
	Non-coding RNAs in metabolic reprogramming	**RNAs:** circ-RNF121, circ-0005963, MALAT1, TUG1, miR-122, miR-105	Induces glycolysis and chemoresist ance; increases HK2, PKM2, LDHA activity with lactate accumulation and proliferation	[60–65]
Exosomal molecules in cancer metabolism (mixed cargo)	Metabolic enzyme and signaling molecule transfer	**Proteins:** PKM2, HK2, LMP1, VEGF, ITGB4, ZIP4	Promotes glycolysis, mitophagy, and metabolic reprogramming in recipient cells	[17, 50, 71–75]
	Hypoxia-driven immunometabolic reprogramming	**RNAs:** miR-23a, miR-142-5p, let-7a, miR-21-3p **Proteins:** IDO	Stabilizes HIF-1α; induces EMT; suppresses CD8^+^ T cell function via IDO activation	[66–70]
	Metabolic enzyme and signaling molecule transfer	PKM2, HK2, LMP1, VEGF, ITGB4, ZIP4	Increased glycolysis; mitophagy induction; metabolic reprogramming in recipient cells	[17, 50, 71–75]
Angiogenesis induction	Exosome-induced vascular growth and remodeling	**RNAs:** circ-0044366, circSHKBP1, miR-21-5p **Proteins:** VEGF, Wnt4, IL-8, MMP-9	Stabilizes VEGF mRNA; drives β-catenin nuclear translocation; increases vascular permeability	[77–80, 84, 85]
	Metabolic support of endothelial cells	**Proteins/Enzymes:** CD39, CD73 **RNAs:** VEGFR mRNA, miR-210	Promotes adenosine production and glycolysis activation in endothelial cells; regulates ATP homeostasis under stress	[52, 81]
Immune regulation by exosomes	Immune cell suppression and immune escape	**Proteins:** PD-L1, FasL, TGF-β1 **RNAs:** miR-1246, HISLA, miR-221-3p	Induces M2 macrophage polarization; suppresses CD8^+^ T cell and DC activity; promotes immune tolerance	[88–98, 101–111]
	TAM-mediated glycolytic and metabolic alterations	**Proteins:** DOCK7, IL-6, VEGF, MyD88 **RNAs:** miR-29a-3p, miR-21-5p	Enhances aerobic glycolysis and lactate accumulation in TAMs; stimulates immunosuppressive cytokine production	[97, 102–110]
Adipose-derived exosomal signaling in tumor metabolism	Tumor metabolism modulation by adipocyte exosomes	**RNAs:** miR-155, miR-23a/b **Proteins:** MTTP, PRAP1	Activates AKT/mTOR signaling; blocks ZEB1; reduces ferroptosis sensitivity and enhances OXPHOS	[3, 59, 112–115]

Abbreviations: CAF: Cancer-associated fibroblast; TGF-β: Transforming growth factor beta; miR: MicroRNA; SNHG3: Small nucleolar RNA host gene 3; TUG1: Taurine upregulated gene 1; CYTB: Cytochrome b; COXI: Cytochrome c oxidase subunit I; HK2: Hexokinase 2; GLUT1: Glucose transporter 1; PKM: Pyruvate kinase M; circRNA: Circular RNA; MALAT1: Metastasis associated lung adenocarcinoma transcript 1; LDHA: Lactate dehydrogenase A; HIF-1α: Hypoxia-inducible factor 1 alpha; EMT: Epithelial–mesenchymal transition; IDO: Indoleamine 2,3-dioxygenase; PKM2: Pyruvate kinase M2; LMP1: Latent membrane protein 1; VEGF: Vascular endothelial growth factor; ITGB4: Integrin beta 4; ZIP4: Zinc transporter ZIP4; ECs: Endothelial cells; CD39/CD73: Ectonucleotidases CD39 and CD73; PD-L1: Programmed death-ligand 1; FasL: Fas ligand; HISLA: Hypoxia-inducible small RNA; DC: Dendritic cells; TAM: Tumor-associated macrophage; DOCK7: Dedicator of cytokinesis 7; IL-6: Interleukin 6; MyD88: Myeloid differentiation primary response 88; MTTP: Microsomal triglyceride transfer protein; PRAP1: Proline-rich acidic protein 1; OXPHOS: Oxidative phosphorylation; AKT/mTOR: Protein kinase B/mechanistic target of rapamycin; ZEB1: Zinc finger E-box binding homeobox 1.

## Exosomes as key players in the metastatic process

Metastasis occurs when cancer cells migrate to favorable environments, resulting in more aggressive cancer forms [116]. Cancer-derived exosomes disrupt endothelial barriers, enhancing their metastatic potential, similar to how cancer cells utilize angiopellosis for extravasation. These exosomes enter the bloodstream at tumor sites, disrupting vascular ECs and promoting angiogenesis and vascular remodeling. They traverse continuous barriers through tight junction-related proteins, suppressing the expression of Kruppel-like factors 2 and 4 (KLF2 and KLF4), and cross barriers via transcytosis, inhibiting the endocytic degradation of exosomes [117, 118].

Systemically, exosomes circulate through blood or lymph to distant sites, conditioning tissues to become more receptive to metastasis. They stimulate vascular ECs, enhancing their proliferation and sprouting to support the formation of new blood vessels [117]. Tumor-derived exosomes induce an EMT, in which epithelial cells lose their defining characteristics and acquire migratory and invasive traits. This process is mediated by exosomal microRNAs that inhibit PTEN, activate the PI3K/AKT pathway, and promote tumor spread and metastasis by repressing E-cadherin through the miR-200 family [117–119]. Additionally, exosomes originating from primary tumors carry enzymes such as heparanase, collagenase, and matrix metalloproteinases that degrade the ECM via TGF-β/Smad signaling, facilitating the intravasation of mesenchymal cells into blood vessels [120]. Both primary and secondary niches supported by exosomes create a conducive environment for metastasis by evading immune responses and aiding invasion [121, 122]. Through alterations in gene expression and cellular behavior, exosomes interact with circulating tumor cells (CTCs), influencing their homing and colonization in pre-metastatic niches [119].

In melanoma, exosomes transfer the mesenchymal–epithelial transition factor protein to progenitor cells, promoting lung metastasis [123]. In pancreatic cancer, exosomes activate hepatic stellate cells in the liver to release TGF-β, attracting specific macrophages and establishing a supportive niche for liver metastasis [124]. Exosomal content also directs cancer cells to specific organs through integrins exhibiting organotropism [125]. Cancer treatments can modify exosome content, further influencing metastatic processes. For instance, chemotherapy-exposed breast cancer cells produce exosomes carrying annexin 6, which trigger the production of C–C motif chemokine ligand 2 and activate monocytes in the lung, promoting a pre-metastatic environment [117]. Furthermore, exosomes contribute to the formation of pre-metastatic habitats by inducing pro-inflammatory responses that recruit MDSCs to distant organs. For example, they attract CCR6+CD4+ Th17+ cells to tumor sites, facilitating proliferation and angiogenesis [116].

Various studies have identified specific exosomal proteins that contribute to metastatic behavior [66, 116]. Matrix metalloproteinase-9, overexpressed in colorectal cancer-derived exosomes, facilitates ECM degradation via TGF-β/Smad signaling activation [126]. Specific markers, such as EGF-like repeats from urinary exosomes, can predict muscle metastasis in bladder cancer patients [127]. Additionally, exosomal miR-21 and the let-7 family modulate critical signaling pathways, including Wnt, Ras, TGF-β, and p53, promoting EMT and enhancing cell migration and invasion [19, 80]. Similarly, exosomal lncRNAs, such as lncRNA-CRNDE-h and lncRNA-H19, are associated with tumor invasion and metastasis by activating pathways that promote growth and vascularization [128–132]. When cancer-derived exosomal microRNA-31, associated with metastasis, is overexpressed, it inhibits local invasion while promoting proliferation and migration. Moreover, MDA-MB-231 cells that have metastasized exhibit elevated levels of miR-130a and miR-328 in their exosomes [133].

### The impact of exosomal cargo on cancer drug resistance

Tumor resistance to treatment presents a significant challenge in oncology, driven by diverse mechanisms that enable tumor cells to evade therapeutic effects. Tumor heterogeneity results in varied cell populations, some of which are inherently resistant to treatments. Cancer cells increase the expression of anti-apoptotic proteins like Bcl-2 to evade apoptosis, while metabolic reprogramming allows them to adapt their survival pathways under therapeutic stress [134–137]. ATP-binding cassette (ABC) transporters actively expel chemotherapeutic agents, reducing drug uptake and limiting intracellular drug availability [16, 138]. Mutations in drug target genes can alter binding affinities, rendering therapies ineffective. Cancer cells’ superior DNA repair capabilities counteract chemotherapy-induced damage, while cancer stem cells (CSCs) promote genetic heterogeneity and intrinsic resistance, driving tumor repopulation [25, 138]. Additionally, epigenetic modifications, such as altered DNA methylation patterns, regulate gene expression associated with drug resistance mechanisms [138]. Exosomal cargo plays a pivotal role in driving cancer drug resistance by enabling intercellular communication and transferring resistance factors. This process disseminates survival signals, contributing to therapy failure and tumor relapse [139, 140].

### Exosomal proteins and RNAs induce drug resistance in tumor cells

Exosomal proteins play a critical role in drug resistance by enhancing survival signaling, facilitating immunological evasion, and promoting angiogenesis. These proteins also affect DNA repair, mitochondrial function, and gene expression, contributing to tumor heterogeneity [46, 66]. Cancer cells utilize exosomes to expel chemotherapy agents, thereby decreasing intracellular drug concentrations and fostering resistance [141]. For instance, squamous cell carcinoma cells in the oral cavity produce cisplatin through small vesicles, leading to both de novo and acquired resistance [142]. Additionally, exosomes facilitate the transfer of resistance traits between cancer cells, disseminating drug-resistant phenotypes throughout the TME [142]. Notably, the X-linked inhibitor of apoptosis protein and Bcl-2, found in exosomes derived from cancer cells, inhibit apoptosis while enhancing cell survival [143].

Exosomes containing glycolytic enzymes are associated with increased drug resistance [72]. They also transport Nrf2, a protein involved in metabolic adaptation in cancer cells, allowing drug-sensitive cells to acquire resistance [144, 145]. Furthermore, exosomes carry PGC-1α, which regulates mitochondrial metabolism and promotes OXPHOS reprogramming, along with Fyn-related kinase (FRK), which enhances cancer cell stemness and resistance [58]. Annexin A3, prevalent in atypical fibroblasts, facilitates metastasis and resistance by transferring exosomes from resistant to susceptible target cells [81]. Exosomal TGF-β has been linked to the dynamic phenotypic plasticity of cancer cells and represents an evasive mechanism that diminishes treatment efficacy. Consequently, targeting TGF-β and its signaling pathway regulators within EVs presents a promising strategy to mitigate the evolution of drug-resistant malignancies [126]. Similarly, CAF-derived exosomes contain VCAM-1, which can activate resistance pathways (AKT and MAPK) in lung cancer cells [146].

CircRNAs and microRNAs within exosomes are pivotal in metabolic reprogramming and therapy resistance [17, 147--149]. In NSCLC, hypoxic exosomes transport PKM2, promoting glycolysis and reprogramming CAFs to create an acidic TME that fosters drug resistance and tumor proliferation [150]. CAF-derived exosomes also deliver microRNAs such as miR-92a-3p, which activate Wnt/β-catenin signaling and inhibit cell death in colorectal cancer [151]. Additionally, miR-98-5p enhances cisplatin resistance by downregulating cell cycle regulators, including CDKN1A and CDKN1B [152]. CAFs transmit the Snail protein via exosomes, promoting hypoxia and increasing resistance by upregulating HIF-1α [153, 154]. In pancreatic cancer, exosomes released following gemcitabine treatment upregulate Snail protein, contributing to resistance to anticancer therapies and tumor proliferation [155].

Furthermore, colon cancer cell-derived exosomes can elevate phosphorylated Akt levels and downregulate PTEN, leading to cetuximab resistance [156]. Gemcitabine-resistant cells produce exosomes containing miRNA-222-3p, which targets SOCS3, thereby increasing treatment resistance and cancer spread. MSC-generated exosomes activate the MEK/ERK pathway, resulting in fluorouracil resistance across various malignancies [157–160]. MicroRNAs such as miR-21, miR-1247-30, miR-155, and miR-210 also contribute to cancer therapy resistance by modulating key signaling pathways (Table 3) [57, 161, 162]. Moreover, exosome-derived miRNAs are implicated in drug resistance in multiple myeloma (MM) through several mechanisms. For instance, miR-1252-5p reduces HPSE expression, enhancing sensitivity to bortezomib (BTZ). miR-140-5p and miR-28-3p from bone marrow stromal cells inhibit SPRED1, activating the MAPK pathway and promoting BTZ resistance. MSC-derived exosomal miR-155 induces stemness in MM cells, leading to chemoresistance. Hypoxia elevates exosomal miR-182, which targets the tumor suppressor SOX6, conferring resistance to carfilzomib. Additionally, decreased levels of exosomal miR-17-5p, miR-20a-5p, miR-15a-5p, and miR-16-5p are associated with BTZ resistance, likely due to disrupted regulation of apoptosis and cell cycle pathways [8–10].

**Table 3:** The impact of exosomal cargo in cancer metastasis and drug resistance

**Role**	**Mechanism**	**Exosomal cargo**	**Cancer types**	**Ref.**
Metastatic niche formation	Prepares distant sites by modulating immune response, enhancing angiogenesis, and ECM remodeling	Proteins: MMPs, heparanase; miRNAs: miR-200 family, miR-21; lncRNAs: H19, CRNDE-h	Breast, melanoma, pancreatic; lung and liver metastasis	[118–123, 129–133]
Epithelial–mesenchymal transition	Induces epithelial cells to gain migratory/invasive properties via gene regulation	miR-200 (suppresses E-cadherin), PTEN inhibitors, PI3K/AKT activators	Breast, colorectal, lung cancer	[117–119]
Organotropism	Directs cancer cells to specific organs through integrins and protein transfer	Integrins, MET protein	Melanoma (lung), pancreatic (liver)	[123–125]
Pro-inflammatory niche formation	Recruits immune cells (MDSCs, Th17) to pre-metastatic sites, promoting proliferation and angiogenesis	Annexin 6, chemokines (CCL2), cytokines	Breast (chemotherapy-modified exosomes)	[116, 117]
Drug efflux and detoxification	Removes chemotherapeutic agents from tumor cells via exosomal export	Chemotherapy drugs (e.g., cisplatin, oxaliplatin)	Oral squamous cell carcinoma, leukemia	[141, 149]
Inhibition of apoptosis	Promotes survival signaling pathways, preventing cell death	Bcl-2, XIAP	Various cancers	[143]
Metabolic reprogramming	Enhances glycolysis and mitochondrial adaptation to sustain drug resistance	PKM2, Nrf2, PGC-1α; circRNA-122	NSCLC, lung, pancreatic cancers	[144–150]
Horizontal transfer of resistance	Spreads resistance traits among cancer cells within tumor microenvironment	miR-222-3p, miR-92a-3p; proteins: Snail, VCAM-1	Pancreatic, colorectal, lung cancers	[151–155]
Activation of oncogenic pathways	Modulates signaling pathways involved in therapy resistance	miR-21, miR- 155, miR-210	Multiple cancer types	[57, 162, 168]
Lipid-mediated drug resistance	Alters membrane rigidity and intracellular drug distribution impacting efficacy	Sphingomyelinase, membrane lipids	Emerging evidence across cancers	[162–164]

Abbreviations: ECM: Extracellular matrix; MDSCs: Myeloid-derived suppressor cells; CRC: Colorectal cancer; NSCLC: Non-small cell lung cancer; SCC: Squamous cell carcinoma; CAF: Cancer-associated fibroblast.

### Exosomal lipids in cancer drug resistance

As key components of the membrane structure, exosomal lipids influence membrane rigidity and the formation of microdomains, which affect the localization and function of membrane proteins, including drug targets. This alteration in lipid composition can reduce drug efficacy [162]. Their transfer can modify lipid metabolism in recipient cells, impacting energy production and contributing to drug resistance. Additionally, exosomal lipids may limit drug access within cells by affecting drug sorting into intracellular spaces, impacting the accessibility of drugs, and halting lysosomal degradation [163]. Furthermore, elevated sphingomyelinase levels in exosomes have been connected to medication resistance, suggesting that metabolic enzymes transferred via exosomes modulate downstream signaling pathways. Exosomal lipids’ role in antitumor resistance research is still in its early stages, but their potential to alter lipid metabolism and modulate TME interactions offers significant opportunities for further study [22, 49, 164].

## Exosomes as therapeutic targets

Cancer treatment is increasingly moving toward personalized therapies to enhance efficacy and minimize side effects [20, 131]. Exosomes, compared to liposomes, demonstrate superior cellular absorption, facilitating effective delivery of functional cargo. Their minimal immune clearance, stability, and nanoscale size position exosomes as exceptional drug carriers, enabling targeted delivery and improving cancer treatment outcomes. As natural transporters, exosomes efficiently deliver CRISPR/Cas9 plasmids to cancer cells, promoting cell death [49]. Milk-derived exosomes, researched for oral chemotherapy delivery, reduce toxicity and improve drug absorption. Additionally, exosomes derived from tumors and DCs, when loaded with antigens, elicit robust immune responses, enhancing T-cell activity and reducing immunosuppressive markers [36, 134].

Engineered exosomes, such as iExosomes, can overcome biological barriers to precisely target oncogenic proteins, delivering siRNA, miRNA, or metabolic modulators that disrupt cancer survival pathways, including those in resistant CNS malignancies [165]. RNA-modified exosomes have been shown to transfer siRNA to malignant cells, improving outcomes in lung cancer [166]. Furthermore, exosomes carrying imatinib or siRNA targeting oncogenes inhibit tumor growth in chronic myeloid leukemia [167, 168]. Exosomes loaded with antagomicroR-222/223 sensitize breast cancer cells to chemotherapy, extend survival in mouse models, and reduce angiogenesis in ovarian tumors by lowering VEGF and HIF-1α levels [67, 169, 170].

Recent advancements include miR-34c-5p carried by exosomes to eliminate CSCs and miR-770 to increase doxorubicin sensitivity in TNBC by modulating apoptosis and the TME. Mesenchymal cell-generated exosomes also sensitize leukemia cells by triggering the caspase pathway to imatinib, offering a good approach for resistant cancers [170]. Patient-specific exosomes can transfer P-gp inhibitors, potentially reversing therapy resistance in tumor cells. Engineered exosomes designed to traverse multiple biological barriers offer promising strategies for targeting resistant CNS malignancies [49].

Conversely, targeting exosomes presents a promising approach to combat antitumor resistance. Inhibition of neutral sphingomyelinase (NSM) with GW4869 has been shown to sensitize cisplatin-resistant ovarian cancer cells [171]. Compounds such as ketotifen and cannabinol reduce exosome secretion, enhancing sensitivi1ty to chemotherapy, while rhamnose-emodin diminishes vesicle release in doxorubicin-resistant malignant cells, reversing drug resistance by suppressing resistance-associated miRNAs [172]. AKT inhibitors have demonstrated efficacy in minimizing exosome-induced chemoresistance [173, 174]. The combination of engineered exosomes with immunotherapy, such as CAR-T or CAR-NK cell-derived exosomes, holds potential for improving cancer treatment outcomes [21]. Despite the promise of CAR-based T-cell adoptive immunotherapy, challenges arise due to the complexity and heterogeneity of cancer. Exosomes derived from CAR-T cells may serve as complementary tools to CAR-T therapy, representing a forward-looking strategy to address current limitations [175, 176]. Future developments may focus on selectively loading mRNA or controlling target gene loading within exosomes, engineering donor cells, modifying exosome surfaces, and employing synthetic nanoparticles. These advancements could significantly enhance precision medicine and gene therapies. Despite challenges such as drug-loading efficiency and stability, exosome-based therapies show promise for tracking tumors and advancing personalized cancer treatment [49, 177, 178].

## Conclusion

Exosomes significantly influence cancer cell metabolism and the surrounding cellular environment by delivering bioactive substances, thereby promoting malignancy. They facilitate the delivery of pro-angiogenic factors, degrade the ECM, and modulate immune responses, contributing to vascularization, invasion, immune escape, and the formation of metastatic niches. Exosomes also play a pivotal role in therapy resistance by carrying drug resistance markers, efflux pumps, and anti-apoptotic factors, underscoring their impact on therapeutic failure and the necessity of targeting exosomal pathways. Given their stability, ability to cross biological barriers, and efficient delivery of functional cargo, exosomes present considerable promise as therapeutic agents. Targeting exosome biogenesis or cargo loading can disrupt tumor pathways, while engineered exosomes enable precise, low-toxicity drug delivery.

## Future perspectives and challenges

Exosome-targeted strategies represent a transformative frontier in cancer medicine, offering innovative solutions for more targeted and personalized therapies. Engineered exosomes exhibit significant potential in addressing cancer drug resistance and reducing drug toxicity. Although their ability to traverse biological barriers makes them ideal for treating metastatic cancer, challenges such as low-yield isolation and purification, lack of standardization, immune clearance, and exosomal heterogeneity remain. Moreover, while preclinical research and early-stage clinical trials have shown promise, substantial scientific and regulatory challenges must be addressed before these discoveries can be translated into widely available therapies. For exosome-based medicines to be effectively integrated into standard cancer treatment protocols, several critical issues must be resolved.
